# Validation of the Organizational-Based General Self-Esteem Scale

**DOI:** 10.3389/fpsyg.2022.865153

**Published:** 2022-06-14

**Authors:** Lorenzo Filosa, Guido Alessandri

**Affiliations:** Department of Psychology, Faculty of Medicine and Psychology, “Sapienza” University of Rome, Rome, Italy

**Keywords:** organizational-based self-esteem, self-esteem at work, scale development, confirmatory factor analysis, measurement invariance, validity and reliability

## Abstract

Using data from four different samples of full-time employees (*N*_total_ = 2,474), the present study was aimed to introduce and demonstrate the validity and reliability of the Organizational-Based General Self-esteem Scale (OB-GSE) a new six-item self-report scale to measure organizational-based self-esteem (OBSE) at work. Results provided evidence of (1) validity (internal, external, and convergent), (2) reliability, and (3) temporal stability of the OB-GSE scale. All in all, results attested the usefulness and the effectiveness of the OB-GSE scale.

## Introduction

Organizational-based self-esteem (OBSE) is defined as “the degree to which an individual believes him/herself to be capable, significant and worthy as an organizational member” (Pierce and Gardner, 2004, p. 593). It represents a specialized (i.e., domain-specific) form of self-esteem capturing the feeling of *being a valuable and effectual member of an organization* (Pierce et al., 1989; Pierce and Gardner, 2004). Thus, OBSE naturally differs from generalized or global self-esteem (GSE) that is instead conceptualized as an individual’s overall self-evaluation *in general* as a person, without referring specifically to any particular area of life (Donnellan et al., 2011).

Research conducted in the organizational setting attested the importance of OBSE in predicting employees’ attitude, behavior, and health (Schaubroeck et al., 2012; Brown and Zeigler-Hill, 2018). For example, meta-analytical findings from Bowling et al. (2010) linked OBSE to key organizational outcomes such as job satisfaction, commitment, and involvement. Other studies have shown significant and positive correlations of OBSE with job performance (Pierce and Gardner, 2004; Bowling et al., 2010), extra-role performance (such as organizational citizenship behaviors), and significant and negative correlations with turnover intentions (Bowling et al., 2010).

All in all, above results agree with basic assumptions derived from general theoretical perspectives focused on the relationships among basic individuals’ characteristics and work attitudes. For example, according to the self-consistency theory (Korman, 1976), people are generally motivated to engage in behaviors that are in line with their perceived self-esteem level, in order to preserve a coherent view of themselves. Accordingly, empirical studies found that employees tend to maintain their self-perception levels by developing positive (or negative) attitudes toward their job in conformity with their positive (or negative) self-view (Pierce et al., 1989). A similar stance is taken by the job demands-resources (JD-R) model (Demerouti et al., 2001), pointing to OBSE as a personal resource strongly associated with work engagement and job performance (Mauno et al., 2007; Bakker and Demerouti, 2008). A result confirmed by several empirical studies.

The idea that OBSE represents one of the most important psychological resources employees can invest to cope with stressors and strains (Hobfoll, 1989; Brown and Zeigler-Hill, 2018; Perinelli et al., 2021) is also strongly advocated by the conservation of resources (COR) theory (Hobfoll, 1989; Hobfoll et al., 2018). According to COR theory, self-esteem represents a key resource for individual adaptability (Hobfoll, 2002). COR theory also states that humans tend to preserve and nourish their self-regard and self-view, because they are vital to overcome the stress caused by threatening situations (Hobfoll et al., 2018), as attested by studies reporting negative correlations of OBSE with depression and physical symptoms (Bowling et al., 2010).

The importance of OBSE and the need of unravelling the processes that link this construct with important organizational processes and outcomes, clearly call for the need of a reliable instrument for assessing it. Usually, in those and many other studies, OBSE has always been measured with the OBSE scale proposed by Pierce et al. (1989), a 10-item self-report scale covering the main features of the construct. The Pierce et al. (1989) scale has several strengths. First, it is brief and very easy to administer. Second, it is high face-valid. Third, commonly reported alpha coefficients have proved to be high. However, psychometric evidence supporting its validity are quite limited. First of all, despite the scale being of common use, to the best of our knowledge, there is no study available in literature which has evaluated its internal validity or factorial structure through explorative or confirmative factor analysis or any other approach. As stated above, only internal consistency (i.e., Cronbach’s alpha) results are often considered in literature.

Clearly, it is worthy to note that there is an issue with evidence supporting the internal validity of many of the most widely used psychological instruments in different areas of psychology (Flake et al., 2017; Hussey and Hughes, 2020). This problem, also named by Hussey and Hughes (2020) the *hidden invalidity* problem, represents a serious threat to the validity of any set of research findings based on the application of an instrument considered the golden standard, because ignoring the internal validity (and, in some instances the actual dimensionality) of a scale can lead to inappropriate measurement of the underlying latent variable (see Rhemtulla et al., 2020). As stated above, the validity of the 10-item OBSE scale introduced by Pierce et al. (1989) has been usually assumed but never deeply investigated. The only investigation we are aware of seemed to suggest that the Pierce et al.’ OBSE scale did not obtain an adequate level of internal validity (see Filosa, 2022), inducing us to suspect the presence of a hidden invalidity problem. Another issue with the Pierce et al. (1989) scale is that items seem generic, and do not refer specifically to organizational aspects of workers’ activity (such as interactions with direct colleagues or inclusion in a working group/team). At the same time, many items are ambiguous and redundant (for instance, an item such as “I am helpful” is very similar to “I am cooperative,” or “I am taken seriously” is very similar to “I am important).

Given the above reasons, in this study, we move from an analysis of the content and the psychometric properties of the Pierce et al. (1989) 10-item scale, and on these bases, we introduce a newly instrument, specifically developed as a reliable and valid measure of OBSE, the Organizational-Based General Self-esteem (OB-GSE). Below we describe the process leading to the development of this new instrument.

### The Development of the OB-GSE

The first step to develop the OB-GSE was to identify a pool of item covering the key feature of the construct, namely being a valuable and effectual member of an organization (see Pierce et al., 1989; Pierce and Gardner, 2004). These items were newly generated, or selected and modified from existing measures of the same or of similar constructs. Most of these items were written as positive statement (but not all). Items assessing the negative pole of the construct were included where necessary. No attempt for balancing negative and positive items was done. The final set of items was submitted for review to two scholars expert in the field, and then further refined. The items then considered for empirical analyses resulted at first glance similar to those of the Pierce et al.’ scale. For instance, items such as “*I feel respected by all my colleagues,*” “*I’m considered an essential part of the workgroup*” and “*I adequately complete assigned duties,*” are strictly comparable with Pierce et al.’ items such as “*I count around here,*” “*I am important,*” or “*I am efficient.*” However, it is worthy to note that the final set of OB-GSE items, although comparable with Pierce et al.’ OBSE scale items, are more specific and non-redundant. That is to say that, an item like “I adequately complete assigned duties” is more specific and more related to a specific work aspect than an item like “I am efficient,” which is generic and not anchored to an actual work aspect.

Furthermore, we decided to adopt the following statement as introduction to the scale: “*In the statements below, you are asked to describe your relationship with your organization with respect to key areas of your organization’s life. There are no right or wrong answers, simply indicate the answer that best reflects your experience.*” Importantly, this introduction is similar to that used for the Pierce et al.’ OBSE scale. In addition to this introduction, items are preceded by the statement “*In my organization*….” The complete response format is a 5-point Likert-type scale (1 = strongly disagree; 2 = disagree; 3 = neither agree nor disagree; 4 = agree; 5 = strongly agree). The approximate duration to fill out the questionnaire is 1 min. All the items are operationalized in the same direction of the construct, so high scores indicate high OBSE levels. The validity of the items has been established trough a consensus panel, where three different experts rated the content of the items for their coherence with the OBSE construct. Full detail on these procedures is offered in Filosa (2022).

### The Present Study

Using data from four different samples of Italian full-time employees, the present study was aimed to introduce and demonstrate the validity and reliability of the OB-GSE, in five analytical steps. As a first step, we investigated the internal validity (through confirmatory factor analysis) and reliability (through both Cronbach’s alpha and omega) of the OB-GSE in all the samples. In this step, we also investigated in parallel the psychometric properties of the Pierce et al.’ scale. As a second step, in order to ascertain the external validity of the new OB-GSE scale, we examined the correlations between OB-GSE and other key organizational variables, using data from Samples 1 to 3. We had some hypotheses on the correlations between OB-GSE and the organizational variables. These hypotheses relied upon previous studies attesting that positive self-evaluations foster work adjustment. This is the case, for example, of OBSE that has been found positively associated with global self-esteem, and feelings of mastery and competence at work (Pierce and Gardner, 2004; Bowling et al., 2010), but negatively associated with levels of neuroticism (Judge et al., 2002; Bowling et al., 2010). In terms of job attitude, OBSE has been linked to job satisfaction (Judge and Bono, 2001; Pierce and Gardner, 2004; Kuster et al., 2013; Orth and Robins, 2022), and work commitment (especially, with affective and normative; Meyer and Allen, 1997; Pierce and Gardner, 2004; Mauno et al., 2006; Bowling et al., 2010). Furthermore, workers’ positive self-evaluations seem to support their sense of engagement with their work (Bakker and Demerouti, 2008), and OBSE, in particular, has been posited as one of the best personal resources in predicting work engagement (Mauno et al., 2006, 2007; Bakker and Demerouti, 2008). At the same time, global self-esteem sustains workers’ well-being (Bowling et al., 2010; Pierce et al., 2016) and prevents job burnout (McMullen and Krantz, 1988; Rosse et al., 1991; Best et al., 2005), stress (Jex and Elacqua, 1999), and depression (Tang and Ibrahim, 1998). Lastly, OBSE is negatively correlated with workers’ intentions to quit with their job (Bowling et al., 2010). Thus, following these results, in this study we expected positive correlations of individuals’ scores on the new OB-GSE with GSE, job satisfaction, commitment, self-efficacy, and work engagement. On the contrary, we expected, negative associations between OB-GSE and neuroticism, burnout, turnover intentions, depression, and perceived stress at work. As a third step, we investigated the convergent validity between our and Pierce et al.’ OBSE scales, using data from Samples 2 to 3. As a fourth step, using data from Sample 3, we analyzed and compared the similarity of the nomological network between of the two measures. As a fifth and last step, we evaluated the degree of stability of the OB-GSE by testing its longitudinal invariance and looking at the test–retest correlation on two-wave data from Sample 4, and then, we moved to test the multigroup measurement invariance across the four samples and between gender.

## Materials and Methods

### Samples

Below we provide a description of each sample included in the present study.^1^

**Sample 1**. Participants were 1,306 (69% males) full-time employees of a national public organization operating in the administrative sector. Age ranged from 18 to 41 years (*M*_age_ = 24.0, SD_age_ = 3.70), while tenure ranged from 1 to 11 years (*M*_tenure_ = 2.55, SD_tenure_ = 2.20).

**Sample 2**. This sample includes 334 (42.5% males) full-time employees of different public and private organizations. Their ages ranged from 18 to 66 years (*M*_age_ = 47.10, SD_age_ = 10.80), while their tenures ranged from 4 to 47 years (*M*_tenure_ = 20.90, SD_tenure_ = 10.69).

**Sample 3**. Participants for this sample were 557 (45.6% males) full-time employees of different public and private organizations. Age ranged from 18 to 67 years (*M*_age_ = 44.70, SD_age_ = 13.64), while tenure ranged from 1 to 42 years (*M*_age_ = 17.31, SD_age_ = 12.53).

**Sample 4**. Participants were 277 (44.4% males) full-time employees of a national public organization operating in the administrative sector. Age ranged from 19 to 67 years (*M*_age_ = 45.23, SD_age_ = 13.81), while tenure ranged from 1 to 11 years (*M*_tenure_ = 18.32, SD_tenure_ = 12.64).

### Procedure

For all of them, procedures were carried out in accordance with the Declaration of Helsinki and were approved by the board of ethics of the Sapienza Department of Psychology. We also followed the same method of recruitment for every sample. Participants were contacted and sent an invitation letter; then, before the survey assessment, participants who agreed to take part in the study were informed about the general topic and purpose of the study, and the possibility to resign participation at any moment. They were also told that participation was voluntary and anonymous, and that their data would be used only for research purposes. Individuals who accepted to participate received a link to fill out the questionnaires online. A summary of the main characteristics of the samples is presented at Table 1.

**Table 1:** Descriptive statistics of study samples.

	Sample 1	Sample 2	Sample 3	Sample 4
*N*	1,306	334	557	277
Gender	Male = 69% Female = 31%	Male = 42.5% Female = 57.5%	Male = 45.6% Female = 54.4%	Male = 44.4% Female = 55.6%
Mean age (SD)	24.0 (3.70)	47.10 (10.80)	44.70 (13.64)	45.20 (13.78)
Mean tenure (SD)	2.55 (2.20)	20.90 (10.69)	17.31 (12.53)	18.32 (12.64)

### Measures

#### Organizational-Based Self-Esteem

The OB-GSE presented in this paper was administered in all the samples. Items from this scale are presented in Table 2. Concurrently, the Pierce et al.’ (1989) OBSE scale was administered only to participants included in Samples 2 and 3 in order to investigate its internal validity as well as the convergent validity between OB-GSE and Pierce et al.’ OBSE scale. Examples of items from this scale are: “I count around here” and “I am helpful.” Both scales included Likert-type items (response scale: 1 = strongly disagree; 5 = strongly agree). Cronbach’s alpha and omega for both OBSE scales are presented in Table 3 and deeply discussed in the Results section.

**Table 2:** Descriptive statistics, factor loadings and item-total scale-score-corrected correlation for each item the OB-GSE in all study samples.

Item	Text	Sample 1 (*α* = 0.81, *ω* = 0.82, AVE = 0.78, CR = 0.93)			Sample 2 (*α* = 0.78, *ω* = 0.80, AVE = 0.65, CR = 0.87)			Sample 3 (*α* = 0.76, *ω* = 0.80, AVE = 0.68, CR = 0.88)			Sample 4 T1 (*α* = 0.78, *ω* = 0.79, AVE = 0.71, CR = 0.90)		
		Mean (SD)	*λ*	*r*_tt_	Mean (SD)	*λ*	*r*_tt_	Mean (SD)	*λ*	*r*_tt_	Mean (SD)	*λ*	*r*_tt_
OB-GSE1	…I’m considered an essential part of the workgroup.	4.27 (0.71)	0.671	0.608	3.92 (0.82)	0.738	0.651	4.13 (0.86)	0.722	0.658	4.25 (0.83)	0.724	0.581
OB-GSE2	…I often have the impression of being left on the sidelines by my workgroup (*reverse scored*).	4.61 (0.63)	0.576	0.525	3.75 (0.99)	0.542	0.467	3.98 (1.02)	0.629	0.522	4.06 (0.98)	0.600	0.501
OB-GSE3	….I adequately complete assigned duties.	4.49 (0.56)	0.512	0.511	4.24 (0.62)	0.534	0.496	4.47 (0.63)	0.320	0.327	4.50 (0.57)	0.446	0.388
OB-GSE4	…I feel completely accepted by my colleagues.	4.27 (0.69)	0.795	0.679	3.86 (0.78)	0.764	0.651	3.95 (0.91)	0.820	0.683	4.10 (0.82)	0.776	0.621
OB-GSE5	…I help colleagues who have heavy workloads.	4.34 (0.63)	0.535	0.476	3.84 (0.74)	0.488	0.434	4.03 (0.88)	0.378	0.353	4.00 (0.78)	0.486	0.340
OB-GSE6	…I feel respected by all my colleagues.	4.20 (0.70)	0.793	0.674	3.84 (0.75)	0.701	0.590	4.00 (0.86)	0.836	0.722	4.04 (0.82)	0.727	0.599

*The introduction to the scale reads “In my organization….” *λ*, factor loadings; *r*_tt_ = item-total scale-score-corrected correlation coefficient; AVE, average variance; CR, composite reliability*.

**Table 3:** Cronbach’s alpha and omega coefficient, with confidence intervals, of study variables across samples.

Variable	Sample 1	Sample 2	Sample 3	Sample 4
OBSE—OB-GSE	0.81 [0.80–0.83]/0.82 [0.81–0.84]	0.78 [0.75–0.82]/0.80 [0.76–0.83]	0.79 [0.75–0.83]/0.80 [0.75–0.85]	0.78 [0.75–0.82]/0.79 [0.75–0.83]
OBSE—Pierce et al. scale		0.86 [0.85–0.88]/0.87 [0.84–0.89]	0.88 [0.87–0.91]/0.89 [0.87–0.91]	
GSE—3-item scale	0.77 [0.74–0.78]/0.79 [0.75–0.81]			
GSE—Lifespan self-esteem scale			0.89 [0.87–0.91]/0.89 [0.87–0.92]	
Work self-efficacy	0.86 [0.84–0.88]/0.86 [0.83–0.88]			
Job satisfaction	–		–	
Life satisfaction			–	
Affective commitment	0.81 [0.78–0.83]/0.85 [0.83–0.87]			
Normative commitment	0.74 [0.72–0.76]/0.78 [0.74–0.80]			
Work engagement	0.86 [0.84–0.88]/0.87 [0.84–0.89]			
Neuroticism	0.90 [0.88–0.91]/0.91 [0.89–0.92]			
Emotional exhaustion	0.87 [0.85–0.89]/0.89 [0.87–0.90]		0.86 [0.85–0.87]/0.89 [0.88–0.90]	
Cynicism	0.71 [0.70–0.73]/0.82 [0.80–0.83]		0.76 [0.72–0.79]/0.83 [0.79–0.86]	
Interpersonal strain	0.90 [0.89–0.91]/0.90 [0.89–0.91]		0.90 [0.89–0.91]/0.91 [0.90–0.92]	
Intention to quit	–			
Depression	0.84 [0.82–0.85]/0.89 [0.88–0.90]			
Perceived stress work			0.91 [0.90–0.92]/0.92 [0.91–0.93]	

*Cronbach’s alphas appear before the forward stash, while omega coefficients appear after the forward stash. Confidence intervals (95%) for both indicators appear in the squared brackets (lower and upper bound, respectively) For job satisfaction, life satisfaction, and intention to quit Cronbach’s alphas and omega coefficients are not available (because they were measured with single-item scales), but the dash indicates when that measurement was used*.

#### Global Self-Esteem

GSE was measured with two different scales. The first one, used in Samples 1 and 2, was the three-item version of the Rosenberg self-esteem scale (Rosenberg, 1965) introduced and validated by Perinelli and Alessandri (2020; see also Perinelli et al., 2021). The items (response scale: 1 = strongly disagree; 5 = strongly agree) assessed individual’s feelings of self-worth and value in general, as a person. Instead, in Sample 3, GSE was measured with the 4-item Lifespan Self-Esteem Scale introduced by Harris et al. (2018; response scale: 1 = strongly disagree; 5 = strongly agree). Cronbach’s alpha and omega (Table 3) were acceptable for both scales.

#### Work Self-Efficacy

The Work Self-Efficacy scale (Alessandri et al., 2015, 2021; response scale: 1 = not well at all; 5 = very well) was used to assess employees’ work self-efficacy beliefs. For each item, employees were asked to rate how well they felt capable of performing the described action or behavior related to their job (e.g., “How well can you make work with a very high precision?,” “How well can you organize your job even in presence of things unforeseen and emergency?”). Cronbach’s alpha and omega (see Table 3) were acceptable.

#### Job and Life Satisfaction

Employees of Sample 1 and 3 evaluated their job satisfaction rating a single item (i.e., “Generally speaking, I am very satisfied with my job”; see Nagy, 2002) with a 7-point Likert scale (1 = strongly disagree; 7 = strongly agree). Likewise, employees of Sample 3 rated their life satisfaction on a single item (i.e., “Overall, I am very satisfied with my life”; see Cheung and Lucas, 2014). Differently from Cheung and Lucas (2014), we adopted the same 7-point Likert response scale to align the satisfaction items with each other.

#### Commitment

Affective (seven items) and normative (five items) commitment were measured with the scale introduced by Allen and Meyer (1990), in Sample 1. The response format was a 5-point Likert scale (1 = strongly disagree; 5 = strongly agree). Examples of items are: “I enjoy discussing my organization with people outside it” for affective commitment; “I think that people these days move from company to company too often” for normative commitment. Reliability (see Table 3) resulted acceptable.

#### Work Engagement

The 9-item Utrecht Work Engagement Scale (UWES-9; Schaufeli et al., 2006) was used to assess employees’ work engagement (response scale: 0 = never; 6 = always) in Sample 1. Examples of items are: “At my work, I feel bursting with energy”; “My job inspires me.” Reliability resulted acceptable (See Table 3).

#### Neuroticism

This personality trait was assessed in Sample 1 with the eight items [e.g., “I often get nervous” and “It’s hard for anything or anyone to make me lose my temper (reverse)”] from the Big Five Questionnaire-2 (BFQ-2; Caprara et al., 2007). The response format was a 5-point Likert scale (1 = very false for me; 5 = very true for me). Reliability (Table 3) was acceptable.

#### Burnout

The three components of job burnout were measured, in Samples 1 and 3, by 17 items (5 items for emotional exhaustion, 5 for cynicism, and 7 for interpersonal strain) drawn from the Maslach Burnout Inventory–General Survey (MBI-GS; Maslach et al., 1996) and from the Interpersonal Strain at Work Scale (ISW; Borgogni et al., 2012). Examples of items are: “I feel emotionally drained from my work” for emotional exhaustion; “I have become less interested in my work since I started this job” for cynicism; “At work, I find myself to be insensitive to other people’s problems.” for interpersonal strain. The response format was a 6-point Likert scale (0 = never; 6 = always). Cronbach’s alpha and omega (Table 3) resulted all acceptable.

#### Intention to Quit

In Sample 1, a single-item scale (i.e., “How likely is it that you will leave your job in the next 12 months?”) drawn from the Michigan Organizational Assessment Questionnaire (response scale: 1 = very unlikely; 5 very likely) was used to assess employees’ intention to quit with their actual job (Cammann et al., 1979, Unpublished manuscript).^2^

#### Depression

The 10-item version (e.g., “I was bothered by things that usually do not bother me” and “I felt lonely”) of the Center for Epidemiologic Studies-Depression scale (CESD-10; response scale: 0 = rarely or none of the time; 3 = most or almost/all the time) was used to measure employees’ depression (Andresen et al., 1994) in Sample 1. Cronbach’s alpha and omega (Table 3) were acceptable.

#### Stress

Perceived stress at work was measured only in Sample 3 with a 10-item version of the scale introduced by Cohen et al. (1983; response scale: 1 = strongly disagree; 5 = strongly agree) adapted to work. Specifically, items were reframed to refer to the work context (e.g., “In the last month, how often have you been upset because of something that happened unexpectedly *at work*?”; “In the last month, how often have you felt nervous and stressed *at work*?”). Cronbach’s alpha and omega (Table 3) were adequate. Due to the modification we introduce, we also analyzed the internal structure of this scale to ascertain its internal validity. CFA fit indices resulted adequate [SB*χ*^2^(35, *N* = 557) = 113.929, *p* < 0.001; SCF = 1.44; CFI = 0.957; TLI = 0.945; RMSEA = 0.064, 90% CI [0.051, 0.077], *p* = 0.04] with factor loadings ranging from 0.58 for item 1 to 0.78 for item 10, with a mean of *M*_factorloadings_ = 0.72 (SD_factorloadings_ = 0.07).

### Analytical Strategy

For testing the structural validity of the OB-GSE and Pierce et al.’ OBSE scales, we used confirmatory factor analyses (CFA). Then, we inspected the reliability of the OB-GSE scale by calculating Cronbach’s alpha, omega coefficient (see McNeish, 2018; Flora, 2020), and item-total scale-score-corrected correlations. We also computed the average variance extracted (AVE; Fornell and Larcker, 1981) and the composite reliability for the OB-GSE. Convergent validity was evaluated by testing the convergence between the OB-GSE and the OBSE scale. Construct validity was further evaluated by looking at the similarity of OB-GSE and OBSE scale nomological network. To this aim, (1) we firstly analyzed correlations between the new OBSE scale and Pierce et al.’ OBSE scales, both specified as latent factors in a CFA model (i.e., we analyzed the correlation between the two latent OBSE factors), and then (2) we analyzed correlations between the two OBSE scales and the above presented external variables, and statistically compared their difference. As a further test of the unique (if any) predictive value of the OB-GSE, we further computed semipartial correlation between the new OB-GSE scale and the external organizational variables, controlling for Pierce et al.’ OBSE scale. Finally, we used two-wave data (collected 1 month apart) to test the longitudinal invariance of OB-GSE in order to ascertain the OB-GSE scale test–retest reliability, and then we tested multigroup measurement invariance across all the four samples and between gender.

### Statistical Analyses

Analyses were conducted using M*plus* 8.30 (Muthén and Muthén, 1998–2018), and *R* 4.1.1 (R Development Core Team, 2016) statistical programs. CFAs were implemented using maximum likelihood estimator robust to non-normality^3^ (M*plus* estimator = MLR). Model fit was evaluated by using the Satorra–Bentler *χ*^2^ scaled statistic (SB*χ*^2^; Satorra and Bentler, 2001), the Comparative Fit Index (CFI), the Tucker–Lewis Index (TLI) and the Root–Mean–Square Error of Approximation (RMSEA). For our purposes, we accepted CFI and TLI values > 0.90 and RMSEA values < 0.08 as indicators of acceptable fit (Kline, 2016). All nested models entailed by the longitudinal and multigroup measurement invariance routine (configural, metric and scalar, see Meredith, 1993) were compared by looking at their differences in the CFI. We retained all constrained models showing a change in ΔCFI ≤ 0.010 (Cheung and Rensvold, 2002; Schmitt and Kuljanin, 2008).

Zero-order and semipartial correlation coefficients were computed using individuals’ mean scale scores. All comparisons among zero-order correlations were carried out following the procedure proposed by Hittner et al. (2003) and implemented with the *R* package *cocor* (Diedenhofen and Musch, 2015).

## Results

Table 1 shows descriptive statistics of each of the four samples.

### Internal Validity and Reliability

Results of the CFAs ran on all the samples data, showed a high level of internal validity for the OB-GSE, with good model fit to the data and adequate factor loadings for each item. Complete CFA results for all the samples are presented in Table 2. Specifically, for Sample 1, the CFA of the OB-GSE resulted in a good model data fit [SB*χ*^2^(9, *N* = 1,306) = 50.859, *p* < 0.001; SCF = 1.51; CFI = 0.971; TLI = 0.951; RMSEA = 0.060, 90% CI [0.044, 0.076], *p* = 0.143]. Factor loadings resulted adequate in size (Table 2), ranging from 0.51 for item 3 to 0.79 for item 4, with a mean of *M*_factorloadings_ = 0.65 (SD_factorloadings_ = 0.13). Similar results were found for Sample 2, with good model fit [SB*χ*^2^(9, *N* = 334) = 27.272, *p* < 0.001; SCF = 1.26; CFI = 0.950; TLI = 0.917; RMSEA = 0.078, 90% CI [0.045, 0.102], *p* = 0.075], and adequate factor loadings, ranging from 0.49 for item 5 to 0.76 for item 4 (*M*_factorloadings_ = 0.63 and SD_factorloadings_ = 0.12). Again, the CFA ran on Sample 3 fitted data well [SB*χ*^2^(9, *N* = 557) = 40.561, *p* < 0.001; SCF = 1.28; CFI = 0.953; TLI = 0.922; RMSEA = 0.079, 90% CI [0.056, 0.105], *p* < 0.05]. Factor loadings were adequate (ranging from 0.32 for item 3 to 0.84 for item 6; *M*_factorloadings_ = 0.62 and SD_factorloadings_ = 0.22). Finally, the structural validity of the OB-GSE was further corroborated in Sample 4, where the hypothesized one factor model estimated at T1 resulted in a good fit [SB*χ*^2^(9, *N* = 277) = 23.693, *p* < 0.001; SCF = 1.15; CFI = 0.948; TLI = 0.914; RMSEA = 0.077, 90% CI [0.040, 0.117], *p* = 0.107], and showed adequate factor loadings (from 0.45 for item 3 to 0.78 for item 4; *M*_factorloadings_ = 0.63 and SD_factorloadings_ = 0.14).

In contrast, results from the CFA of Pierce and colleagues’ OBSE scale fared less well. In Sample 2 [SB*χ*^2^(35, *N* = 334) = 276.126, *p* < 0.001; SCF = 1.46; CFI = 0.714; TLI = 0.632; RMSEA = 0.144, 90% CI [0.128, 0.160], *p* < 0.01] and 3 [SB*χ*^2^(35, *N* = 557) = 553.958, *p* < 0.001; SCF = 1.42; CFI = 0.710; TLI = 0.627; RMSEA = 0.163, 90% CI [0.151, 0.175], *p* < 0.01], the hypothesized one factor model fitted the data very poorly. This poor fit can be likely explained by a significant degree of residual correlations attested by modification indices provided by M*plus*.^4^

Turning to the reliability of the OB-GSE, results of Cronbach’s alphas (*M* = 0.79; SD = 0.01; MIN = 0.78 Sample 4, MAX = 0.81 Sample 1), omega coefficients (*M* = 0.80; SD = 0.01; MIN = 0.79 Sample 4, MAX = 0.82 Sample 1), and item-total scale-score-corrected correlation coefficients (see Table 2) pointed to the high level of reliability the OB-GSE scale. In addition, both AVE and composite reliability resulted adequate (see Table 2), ranging from 0.65 to 0.78 and from 0.87 and 0.93, respectively.

### External Validity

Tables 4 and 5 present correlations between OB-GSE and the key organizational variables included in Samples 2 and 3 data, respectively. In general, results revealed that all the correlations were in the expected direction and moderate-to-high in magnitude. Specifically, the correlation between OB-GSE and GSE was positive and moderate-high in both samples (*r* = 0.544 and *r* = 0.333 for Samples 2 and 3, respectively). In addition, the correlation with work self-efficacy from Sample 2 was high and positive (*r* = 0.595) and mimicked those with GSE. On the same line, the associations between OB-GSE and job aptitudes resulted positive and moderate to high: correlations with job satisfaction ranged from *r* = 0.353 (Sample 3) to *r* = 0.565 (Sample 2), while those with commitment in Sample 2 resulted high for affective commitment (*r* = 0.516) and moderate for normative commitment (*r* = 0.266). The correlation with life satisfaction in Sample 3 resulted in the same direction, but smaller in magnitude (*r* = 0.194). Instead, work engagement resulted positively and highly correlated with OBSE (*r* = 0.578) in Sample 2. Turning to burnout, results revealed a similar (but negative) pattern of associations compared to that with work engagement. In particular, for Sample 2, all the components of burnout, namely emotional exhaustion (*r* = −0.510), cynicism (*r* = −0.497), and interpersonal strain (*r* = −0.531) resulted negatively and highly associated with OBSE. For Sample 3, correlations with burnout resulted similar but slightly lower than those of Sample 2 (*r* = −0.208, *r* = −0.373, and *r* = −0.390 for emotional exhaustion, cynicism, and interpersonal strain respectively). Even neuroticism resulted negatively and highly correlated with OBSE (*r* = −0.520) in Sample 2. Finally, intention to quit (*r* = −0.242) and depression (*r* = −0.323) in Sample 2, as well as perceived stress at work (*r* = −0.302) in Sample 3, resulted negatively and moderately associated with OBSE.

**Table 4:** Zero-order correlations between OB-GSE and Sample 1 variables.

Sample 1 variables	(1)	(2)	(3)	(4)	(5)	(6)	(7)	(8)	(9)	(10)	(11)	(12)
1. OB-GSE	–											
2. GSE	0.544	–										
3. WSE	0.595	0.431	–									
4. Job Sat.	0.565	0.535	0.369	–								
5. Aff. Com.	0.516	0.421	0.368	0.581	–							
6. Norm. Com.	0.266	0.229	0.192	0.373	0.423	–						
7. WE	0.578	0.499	0.481	0.670	0.585	0.345	–					
8. Neur.	−0.520	−0.478	−0.416	−0.452	−0.314	−0.164	−0.470	–				
9. Emo. Ex.	−0.510	−0.395	−0.387	−0.524	−0.357	−0.191	−0.613	0.567	–			
10. Cynicism	−0.497	−0.427	−0.345	−0.563	−0.486	−0.251	−0.599	0.537	0.659	–		
11. Int. Strain	−0.531	−0.439	−0.353	−0.471	−0.386	−0.214	−0.509	0.570	0.578	0.688	–	
12. Int. to Quit	−0.242	−0.279	−0.158	−0.380	−0.383	−0.180	−0.307	0.278	0.258	0.409	0.326	
13. Depression	−0.323	−0.304	−0.273	−0.263	−0.180	−0.028	−0.330	0.484	0.478	0.453	0.402	0.280

*OB-GSE, organizational-based general self-esteem; GSE, general self-esteem; WSE, work self-efficacy; Job. Sat., job satisfaction; Aff. Com., affective commitment; Norm. Com., normative commitment; WE, work engagement; Neur., Neuroticism; Emo. Ex., emotional exhaustion; Int. Strain, interpersonal strain; Int. to Quit, intention to quit*.

*All correlations are significant for *p* < 0.001*.

**Table 5:** Zero-order correlations and comparison between OB-GSE, Pierce et al. OBSE scale and Sample 3 variables.

Sample 3 variables	*r* for OB-GSE	*r* for Pierce et al. scale	Semipartial *r* correlation for OB-GSE (controlling for Pierce et al. scale)	Comparison between correlations (Hittner et al., 2003)
GSE	0.333^***^	0.306^***^	0.198^***^	*Z* = 0.89, *p* = 0.37
Job satisfaction	0.353^***^	0.288^***^	0.229^***^	*Z* = 2.14, *p* < 0.05
Life satisfaction	0.194^**^	0.148^*^	0.132^*^	*Z* = 1.45, *p* = 0.15
Burnout (total score)	−0.350^***^	−0.279^***^	−0.239^***^	*Z* = −2.33, *p* < 0.05
Emotional exhaustion	−0.208^***^	−0.200^***^	−0.113^*^	*Z* = −0.25, *p* = 0.80
Cynicism	−0.373^***^	−0.307^***^	−0.240^***^	*Z* = −2.19, *p* < 0.05
Interpersonal strain	−0.390^***^	−0.217^***^	−0.324^***^	*Z* = −5.67, *p* < 0.01
Perceived stress work	−0.302^***^	−0.246^***^	−0.201^***^	*Z* = −1.81, *p* = 0.07

* *p < 0.05*,

** *p < 0.01*,

*** *p < 0.001*.

### Convergent Validity

Results from the CFAs^5^ including both the OB-GSE and Pierce et al.’ OBSE scale (specified as latent factors) revealed that the latent correlations between the two scales results very high. Specifically, they were *r* = 0.711 for Sample 2 and *r* = 0.704 for Sample 3, attesting a high level of convergence between the two scales.

### Nomological Network Across OBSE Scales

Table 5 presents correlations between the OB-GSE and the Pierce et al.’ scale on the one hand, and the organizational variables included in the Sample 3 data on the other hand, semipartial correlations for OB-GSE controlling for Pierce et al.’ scale, and results for the comparison between the correlations. Results from this set of analyses revealed that individuals’ OB-GSE scale scores remained significantly associated with the organizational variables even after controlling for the shared covariance with Pierce et al.’ scale. In addition, the comparison between the dependent overlapping correlations (Table 5) showed significant differences in the size of the correlations of OB-GSE with job satisfaction, burnout (total score), cynicism, and interpersonal strain that resulted higher than the same correlations estimated for the Pierce et al.’ OBSE scale. The remaining four correlations, with GSE, life satisfaction, emotional exhaustion, and perceived stress at work, were not significantly different.

### Temporal Stability

In order to ascertain the temporal stability of our scale, we analyzed the longitudinal measurement invariance and test–retest correlations using two-wave data from Sample 4. As presented in Table 6, longitudinal measurement invariance results revealed that the configural, metric, and scalar invariance models fitted data well, and did not differ significantly from each other, suggesting that a good level of measurement invariance across time was reached. Furthermore, the high test–retest correlation across waves (*r* = 0.673) suggested a good level of stability across time.

**Table 6:** Longitudinal measurement invariance of OB-GSE across waves 1 and 2.

Model	*χ* ^2^	*df*	SCF	CFI	RMSEA	CI 90%	ΔCFI
Only wave 1	23.693^**^	9	1.153	0.948	0.077^n.s.^	[0.040, 0.115]	–
Only wave 2	24.834^**^	9	1.331	0.964	0.080^n.s.^	[0.043, 0.118]	–
Configural	89.490^**^	47	1.161	0.960	0.057^n.s.^	[0.039, 0.075]	–
Metric	94.172^**^	52	1.162	0.961	0.054^n.s.^	[0.036, 0.071]	−0.001
Scalar	101.971^**^	57	1.147	0.958	0.053^n.s.^	[0.036, 0.070]	−0.003

* *p* *< 0.05 and*

** *p < 0.01*,

**χ*^2^, Satorra–Bentler Chi-square statistic; *df*, degree of freedom; SCF, scaling correction factor; CFI, comparative fit index; RMSEA, root mean square error of approximation; CI 90%, RMSEA confidence interval 90%; ΔCFI, CFI difference; and n.s., not significant*.

### Multigroup Measurement Invariance

As last analysis, we examined measurement invariance across the four different samples, and between gender. Results presented in Tables 7 and 8 show that the configural, metric, and scalar (partial) invariance models fitted data well, and did not differ significantly from each other, suggesting that a good level of measurement invariance was reached across both the four samples and gender.

**Table 7:** Multigroup Measurement Invariance of OB-GSE across all samples.

Model	*χ* ^2^	*df*	SCF	CFI	RMSEA	CI 90%	ΔCFI
Configural	153.193^**^	36	1.233	0.957	0.073^**^	[0.062, 0.085]	–
Metric	185.372^**^	51	1.233	0.951	0.066^**^	[0.056, 0.076]	−0.006
Scalar	498.105^**^	66	1.196	0.841	0.104^**^	[0.096, 0.113]	−0.110
Scalar partial	224.930^**^	64	1.190	0.941	0.064^**^	[0.055, 0.074]	−0.010

* *p < 0.05*,

** *p < 0.01*,

*** *p > 0.05*.

*For the scalar partial level of invariance, we had to free the intercept of OB-GSE2 and OB-GSE5 of Sample 1. The ΔCFI for the scalar partial level of invariance is obtained from comparison with the CFI of the metric level of invariance. *χ*^2^, Satorra–Bentler Chi-square statistic; *df*, degree of freedom; SCF, scaling correction factor; CFI, comparative fit index; RMSEA, root mean square error of approximation; CI 90%, RMSEA confidence interval 90%; ΔCFI, CFI difference*.

**Table 8:** Multigroup measurement invariance of OB-GSE across gender.

Model	*χ* ^2^	*df*	SCF	CFI	RMSEA	CI 90%	ΔCFI
Only male	42.997^**^	9	1.205	0.965	0.068^n.s.^	[0.049, 0.090]	–
Only female	55.965^**^	9	1.195	0.929	0.091^**^	[0.069, 0.114]	–
Configural	98.910^**^	18	1.200	0.950	0.079^**^	[0.064, 0.094]	–
Metric	107.242^**^	23	1.207	0.948	0.071^**^	[0.058, 0.085]	−0.002
Scalar	156.325^**^	28	1.173	0.921	0.080^**^	[0.068, 0.092]	−0.024
Scalar partial	118.105^**^	27	1.177	0.944	0.068^**^	[0.056, 0.081]	−0.014

* *p* *< 0.05 and*

** *p < 0.01*.

*For the scalar partial level of invariance, we had to free the intercept of OB-GSE1. The ΔCFI for the scalar partial level of invariance is obtained from comparison with the CFI of the metric level of invariance. *χ*^2^, Satorra–Bentler Chi-square statistic; *df*, degree of freedom; SCF, scaling correction factor; CFI, comparative fit index; RMSEA, root mean square error of approximation; CI 90%, RMSEA confidence interval 90%; ΔCFI, CFI difference; and n.s., not significant*.

## Discussion

The present multi-sample study was aimed to introduce the OB-GSE, a new measure to assess OBSE in the organizational settings. All in all, using data from four different sample of full-time employees, results demonstrated the good psychometric properties of this new scale. Specifically, the multiple CFAs ran on each sample provided evidence for the internal validity of the OB-GSE, with good model fit to the data and appropriate factor loadings for each of the six items composing the scale. Similarly, all the computed reliability coefficients, namely Cronbach’s alpha, omega, and item-total scale-score-corrected correlations, resulted adequate, suggesting good reliability levels for our scale. In addition, AVE and composite reliability resulted adequate.

Furthermore, the associations between OB-GSE and the key organizational variables provided evidence for the external validity of our OBSE scale. The positive correlations with GSE, work self-efficacy, job aptitudes (i.e., satisfaction and commitment), life satisfaction, and the negative correlations with neuroticism, burnout, intention to quit, depression, and stress are in line with previous findings (see Bakker and Demerouti, 2008; Bowling et al., 2010 and Brown and Zeigler-Hill, 2018). Moreover, these results not only further attested the usefulness of the OB-GSE, but also reiterate the important role played by OBSE as a key resource fostering employees well-being and performance.

Additionally, the convergent validity of the OB-GSE has been proven by (1) the correlations with the Pierce et al.’ OBSE scale, which resulted very high in both Samples 2 and 3 data, and (2) by the semipartial correlations with the organizational variables, which remained significant and relatively similar to the paired non-semipartial correlations even after controlling for the shared covariance with the Pierce et al.’ scale. In addition, four external correlations out of 8 for the OB-GSE resulted significantly higher than those of Pierce et al.’ OBSE scale. Although we are aware that these results need to be further explored and replicated, we believe that, taken together with the semipartial correlation results, these findings suggest that the OB-GSE seems to be able to catch a higher proportion of covariance with important organizational variables, actually providing an incremental advantage over the other OBSE scale. Finally, measurement invariance results provided by two-wave data from Sample 4, along with the multigroup invariance among samples and gender, attested a good level of measurement invariance. These results, along with the high test–retest correlation, suggests a high degree of temporal stability for the OB-GSE and invariance among groups. Thus, researcher can confidently use the OB-GSE scale in longitudinal studies at work.

### Implications, Limitations, and Future Directions

All in all, results provided by the present multi-sample study attest the usefulness and the effectiveness of the OB-GSE. The new proposed scale has been proven to be valid and reliable, stable over time and among different groups, brief and easy to administer, and also able to capture a higher proportion of covariance with some organizational variables compared to the classical OBSE scale proposed by Pierce et al., thus representing a solid alternative to this latter scale. Therefore, we believe that the new OBSE scale has the potential to enhance future organizational research on OBSE, especially in long surveys and longitudinal designs.

Although the present multi-sample study provided evidence for the usefulness of the OB-GSE scale, there are still some limitations to note. The present study relied only on data form permanent workers. Future research must explore the usefulness of the OB-GSE scale by testing its psychometric properties on data from workers with temporary contracts, along with measurement invariance among permanent and temporary workers. Another potential limitation regards the time lag between *T*1 and *T*2 of longitudinal measurement invariance. Although longitudinal measurement invariance was well established, future research should ascertain the temporal stability of the scale by inspecting measurement invariance among longer time lag assessment (i.e., many months or years). Future study should also analyze the correlations between the OB-GSE and other important self-esteem scales, such as for example the work-contingent self-esteem scale (Ferris et al., 2009). At the same time, to extend the external validity of the OB-GSE scale, future research should examine the correlations (as well as the predictive effects) with objective or other-report measures like performance or physiological outcomes (i.e., cortisol, HRV), since the present study relied only on self-report measures.

### Considerations on Pierce et al. OBSE Scale

According to our results, Pierce et al.’ scale failed to show an adequate degree of structural validity in both the CFAs we ran on Samples 2 and 3 data, showing poor fit to the data. This problem was probably attributable to a high level of content overlap among items included in this scale. As mentioned above, the modification indices provided by M*plus* suggested high correlations between some items’ residuals. In fact, analyzing and comparing the wording of these items (“I count around here” with “I am taken seriously” and “I am important”; “I can make a difference” and “I am valuable”; “I am helpful” with “I am cooperative”), it is clear that their content overlap. This *content* or *item redundancy* (Boyle, 1991; see also Reise et al., 2018) has been proven to be a cause of correlated residuals (Bandalos, 2021). Consequently, considering that these items appear to be very similar or even synonymous with each other, it cannot be excluded that the high level of internal consistency between items (in this study *α* = 0.86 and *ω* = 0.87 for Sample 2, and *α* = 0.88 and *ω* = 0.89 for Sample 3) and the above-mentioned low internal validity are due to methodological biases in the item-wording that may limit the effectiveness of the scale (Boyle, 1991; Podsakoff et al., 2012; Bandalos, 2021). That said, we encourage researcher to conduct further studies on the dimensionality and the psychometric properties of Pierce et al. OBSE scale in order to replicate our results.

## Conclusion

The present multi-sample study introduces the OB-GSE, a new reliable and valid scale to measure OBSE. All in all, results provided evidence for its reliability, internal and external validity, convergent validity with the classical OBSE scale proposed by Pierce et al. (1989), as well as temporal stability. With the present results, our study introduces a highly valid and reliable tool for measuring a key employees’ personal resource, in line with classical operationalization of organizational-based self-esteem. We hope that it can contribute to the enlargement of this important field of research.

## Data Availability Statement

The datasets supporting the findings of this article will be made available by the corresponding author(s) upon reasonable request.

## Ethics Statement

The studies involving human participants were reviewed and approved by Sapienza Department of Psychology Internal Review Board. The patients/participants provided their written informed consent to participate in this study.

## Author Contributions

LF and GA conceived the study, collected the data, and drafted the manuscript. LF ran analyses. All authors contributed to the article and approved the submitted version.

## Funding

This paper was supported by two grants: Bando Avvio alla ricerca (D.R.n.1258/2021 - Prot. n. 36805 del 7/05/2021) from Sapienza University of Rome, awarded to Lorenzo Filosa; and grant number RG11816433CBD8D3 from Sapienza University of Rome, awarded to Guido Alessandri.

## Conflict of Interest

The authors declare that the research was conducted in the absence of any commercial or financial relationships that could be construed as a potential conflict of interest.

## Publisher’s Note

All claims expressed in this article are solely those of the authors and do not necessarily represent those of their affiliated organizations, or those of the publisher, the editors and the reviewers. Any product that may be evaluated in this article, or claim that may be made by its manufacturer, is not guaranteed or endorsed by the publisher.

## Footnotes

^1^For each sample, we checked for potential careless responses and then excluded those participants (1) whose responses were too fast (i.e., questionnaire completion time less than two standard deviations below the mean), or participants (2) who failed to correctly fill out two check attention questions.

^2^Cammann, C., Fichman, M., Jenkins, D., and Klesh, J. (1979). The Michigan organizational assessment questionnaire (Unpublished manuscript). Ann Arbor, MI: University of Michigan.

^3^The use of a robust estimator was due to the non-normal distribution of the items. Results about univariate skewness and kurtosis for each item, as well as a test of multivariate normality for all the items are reported in Supplementary Table S1 of Supplementary Materials.

^4^For Sample 2, the correlation between the residuals of item 1 and item 3 showed a modification index of 80.078, that of item 1 and item 2 a modification index of 33.713, while that of item 6 and 7 of 38.713; moreover, for Sample 3, the correlation between the residuals of item 1 and item 3 showed a modification index of 116.895, that of item 1 and item 2 a modification index of 71.276, that of item 6 and 7 of 59.618, and that of item 8 and item 10 of 42.154. It is worthy to note that almost the same item residuals resulted highly correlated among them (i.e., item 1 with item 2 and 3, and item 6 with item 7) across Sample 2 and 3. It is noteworthy to report that the Pierce and colleagues’ OBSE scale fitted data very poorly even after freeing the above-mentioned correlation between the residuals [SB*χ^2^*(35, *N* = 334) = 131.366, *p* < 0.001; SCF = 1.41; CFI = 0.882; TLI = 0.834; RMSEA = 0.096, 90% CI [0.080, 0.114], *p* < 0.01] and [SB*χ^2^* (35, *N* = 557) = 324.736, *p* < 0.001; SCF = 1.37; CFI = 0.836; TLI = 0.770; RMSEA = 0.128, 90% CI [0.116, 0.141], *p* < 0.01] for Sample 2 and 3, respectively.

^5^Considering the poor fit obtained by the Pierce et al.’ OBSE scale, for both CFAs, we had to free at least three residual correlations of the Pierce et al.’ OBSE scale to obtain acceptable fit to the data for these models.
