# Supplementary material for: Validation of the Organizational-Based General Self-Esteem Scale
Source: Front Psychol. 2022 Jun 14;13:865153. doi: 10.3389/fpsyg.2022.865153 (PMC9281545; doi:10.3389/fpsyg.2022.865153)
Supplement: Supplementary file 1 [file Table_1.DOCX]

**SUPPLEMENTARY MATERIALS**

Validation of the Organizational-Based General Self-Esteem (OB-GSE) Scale

**Table S1**

*Univariate and Multivariate Skewness and Kurtosis of the OB-GSE scale items.*

|  | Skewness | | | |  | Kurtosis | | | |
| --- | --- | --- | --- | --- | --- | --- | --- | --- | --- |
| Sample | 1 | 2 | 3 | 4 |  | 1 | 2 | 3 | 4 |
| OB_GSE1 | -.73 | -.62 | -.98 | -1.07 |  | .47 | .61 | 1.01 | 1.39 |
| OB_GSE2 | -1.38 | .12 | -.13 | -.28 |  | 1.45 | -.98 | -1.38 | -1.27 |
| OB_GSE3 | -.61 | -.44 | -.67 | -.60 |  | -.20 | .50 | -.22 | -.64 |
| OB_GSE4 | -.73 | -.61 | -.68 | -.76 |  | .70 | .71 | .38 | .62 |
| OB_GSE5 | -.57 | -.55 | -1.02 | -.73 |  | .23 | .88 | 1.79 | .99 |
| OB_GSE6 | -.59 | -.47 | -.84 | -.90 |  | .44 | .65 | .99 | 1.29 |
|  | Multivariate Skewness | | | |  | Multivariate Kurtosis | | | |
|  | 6.55* | 5.61* | 8.37* | 9.71* |  | 72.18* | 64.32* | 70.85* | 67.48* |

* *p* < .05
